# Regional Association Analysis of MetaQTLs Delineates Candidate Grain Size Genes in Rice

**DOI:** 10.3389/fpls.2017.00807

**Published:** 2017-05-29

**Authors:** Anurag V. Daware, Rishi Srivastava, Ashok K. Singh, Swarup K. Parida, Akhilesh K. Tyagi

**Affiliations:** ^1^National Institute of Plant Genome ResearchNew Delhi, India; ^2^Division of Genetics, Indian Agricultural Research InstituteNew Delhi, India; ^3^Department of Plant Molecular Biology, University of Delhi South CampusNew Delhi, India

**Keywords:** grain size, grain weight, metaQTL, QTL mapping, regional association mapping, rice

## Abstract

Molecular mapping studies which aim to identify genetic basis of diverse agronomic traits are vital for marker-assisted crop improvement. Numerous Quantitative Trait Loci (QTLs) mapped in rice span long genomic intervals with hundreds to thousands of genes, which limits their utilization for marker-assisted genetic enhancement of rice. Although potent, fine mapping of QTLs is challenging task as it requires screening of large number of segregants to identify suitable recombination events. Association mapping offers much higher resolution as compared to QTL mapping, but detects considerable number of spurious QTLs. Therefore, combined use of QTL and association mapping strategies can provide advantages associated with both these methods. In the current study, we utilized meta-analysis approach to identify metaQTLs associated with grain size/weight in diverse Indian *indica* and aromatic rice accessions. Subsequently, attempt has been made to narrow-down identified grain size/weight metaQTLs through individual SNP- as well as haplotype-based regional association analysis. The study identified six different metaQTL regions, three of which were successfully revalidated, and substantially scaled-down along with *GS3* QTL interval (positive control) by regional association analysis. Consequently, two potential candidate genes within two reduced metaQTLs were identified based on their differential expression profiles in different tissues/stages of rice accessions during seed development. The developed strategy has broader practical utility for rapid delineation of candidate genes and natural alleles underlying QTLs associated with complex agronomic traits in rice as well as major crop plants enriched with useful genetic and genomic information.

## Introduction

The major goal of contemporary molecular breeding is to understand complex inheritance pattern and genetic basis of agronomically important traits for rapid genomics-assisted crop improvement. Quantitative trait loci (QTLs) mapping is the most commonly adopted strategy for identifying QTLs (a genomic region with one or multiple genes) regulating different complex traits of agronomic importance in crop plants. Till date, genome scans for QTLs have been conducted for variety of agronomic traits in most of the major food crops, which resulted in identification of numerous QTLs^1,2^. However, mere detection of QTLs is usually insufficient for their utilization in crop improvement programs. This is mainly because, confidence intervals for QTLs identified primarily by QTL mapping usually span tens of map units (cM) or hundreds of genes making it difficult to introgress these large genomic regions (QTL intervals) for genetic enhancement of elite crop varieties. Therefore, fine mapping and subsequent map-based cloning of these large QTL genomic intervals to one or few genes responsible for observed phenotypic trait variation are essential. However, fine mapping/positional cloning studies to scale-down a QTL region to candidate genes require development, maintenance and precise multi-location/year field phenotyping of large size (thousands of mapping individuals) advanced generation back-cross and/or RIL (recombinant inbred line) mapping populations. Further, it also requires screening of thousands of mapping individuals from developed mapping populations to identify suitable recombination events within trait-associated QTL intervals. Consequently, fine mapping of these QTLs into potential genes influencing phenotypic trait variation in crop plants requires considerable cost, labor, time and resources. This ultimately impedes fine mapping of majority of the identified QTLs into single gene and essentially affects efficient utilization of identified QTLs for crop improvement.

Association mapping is a proficient complementary approach to QTL mapping for rapid identification of genomic regions (genes) associated with complex quantitative traits. This strategy takes advantage of numerous historical recombinations accumulated within natural germplasm accessions during evolution/domestication and therefore, usually offers a much higher resolution as compared to QTL mapping (Mackay et al., 2009; Gupta et al., 2014). Although first introduced in humans, the utility of association mapping (including candidate gene-based, regional and genome-wide) is now well-demonstrated in multiple diploid as well as complex polyploid crop species to identify trait-associated genomic loci (genes/alleles). Since its inception, association mapping studies have contributed tremendously to our understanding on genetic architecture of complex agronomic traits (Aranzana et al., 2005; Ehrenreich et al., 2009; Meijón et al., 2014; Si et al., 2016; Song et al., 2016). Association mapping is found proficient to rapidly detect more number of natural allelic variations from germplasm accessions and thereby offers much higher resolution as compared to QTL mapping scanned on bi-parental mapping population. However, this strategy detects considerable number of false-positive marker trait associations due to presence of population structure among germplasm accessions used for association analysis (Breseghello and Sorrells, 2006; Sneller et al., 2009). This constraint of spurious marker-trait association can be addressed to certain extent by considering the significant effects of population structure in diverse statistical models as well-adopted by previous association mapping studies in crop plants (Kujur et al., 2015; Upadhyaya et al., 2015, 2016; Bajaj et al., 2016). Despite these efforts, association mapping result still suffers from significant degree of confounding due to strong population structure and cryptic relatedness among accessions used for association analysis. (Brachi et al., 2010). Recently, the process of complex trait dissection in crops has been accelerated due to advent of joint linkage-association analysis and/or QTL region-specific association analysis, which combines the advantages accompanied with both QTL mapping (efficiently detects highly authentic QTLs) and association mapping (higher resolution). The efficacy of this integrated strategy to scale-down and for subsequent fine mapping of the QTL regions into functionally relevant potential candidate genes and natural alleles explaining majority of the quantitative observed phenotypic trait variation in crop plants is well-documented (Lu et al., 2010; Saxena et al., 2014; Zhang et al., 2014; Kujur et al., 2015; Mammadov et al., 2015; Li et al., 2016).

Rice is one of the most consumed cereal food crop in the world and therefore, possesses huge socio-economic relevance. Grain size [grain length (GL) and grain width (GWi)] is one of the most important determinant of grain weight (GWt) and grain yield in rice, besides serving as a vital determinant of consumer preference especially for Basmati trade and commerce. Globally, more than 400 major QTLs regulating grain size/weight have been identified so far, predominantly by traditional linkage/QTL mapping approach and in few cases by genome-wide association studies (GWAS) using diverse bi-parental mapping populations and natural germplasm accessions, respectively (Fan et al., 2006; Zhao et al., 2011; Luo et al., 2013; Zuo and Li, 2014; Si et al., 2016). Genes underlying many of these major QTLs have been subsequently cloned and functionally characterized through fine-mapping/map-based cloning and various functional genomic strategies (Song et al., 2007; Shomura et al., 2008; Mao et al., 2010; Ishimaru et al., 2013). Although many vital QTLs/genes (for instance, *GS3*, *GS5*, *qGL3*, *GW2*, *GW5*, *GW8*, and *TGW6*, etc.) regulating grain size/weight are now well-known; however, majority of these QTLs/genes (except few like *GS3*) have failed to precisely explain grain size/weight trait variation existing within Indian *indica* and aromatic germplasm accessions of rice (Anand et al., 2013; Dixit et al., 2013; Lu et al., 2013). This can be attributed to disparities in genetic constitution of Indian *indica* and aromatic rice accessions from the Chinese *indica* and *japonica* accessions that have been used primarily for mapping of these known cloned QTLs/genes in rice. Meanwhile, many significant efforts have been made to elucidate the genetic basis of grain size/weight variability present within Indian *indica* and aromatic rice accessions by QTL mapping that led to identification of multiple long grain size/weight QTL intervals on different rice chromosomes (Amarawathi et al., 2008; Guleria et al., 2012; Marathi et al., 2012; Swamy et al., 2012; Shanmugavadivel et al., 2013; Vemireddy et al., 2015). Unlike other globally identified and well-dissected major grain size/weight QTLs, genes underlying these major QTLs have not been delineated yet. This has eventually hindered utilization of these QTLs in marker-assisted selection (MAS) for grain size/weight and grain yield improvement of elite Indian *indica* and aromatic rice accessions.

In recent years, substantial progress has been made toward cataloging and characterizing vast number of available rice germplasm accessions. This has constituted phenotypically rich and genotypically diverse genetic resources comprising core/mini-core germplasm accessions and user-specific association panels (Ebana et al., 2008; Li et al., 2010; Ali et al., 2011; Zhang et al., 2011; Tiwari et al., 2015; International Rice Gene Bank^3^). Exploitation of these diverse genetic resources for various high-throughput genetic analysis and genomics-assisted crop improvement of rice is now conceivable with advent of next-generation sequencing (NGS)-led structural, functional and comparative genomic strategies. Numerous (Millions) sequence variants including SNPs (single nucleotide polymorphisms) and InDels (insertions–deletions) for thousands of resequenced rice accessions belonging to different *indica*, *japonica*, *aus*, aromatic and wild populations are available at a genome-wide scale in public-domain (Youens-Clark et al., 2010; Zhao et al., 2014; Alexandrov et al., 2015; McCouch et al., 2016). Accessibility to whole genome resequencing- and array-based SNP genotyping data for large number of diverse accessions provides unique opportunity in utilizing this information for regional association analysis of known QTL intervals to expedite their validation and delineation of underlying genes in rice (Chen et al., 2014; Zhao et al., 2014; Singh et al., 2015; McCouch et al., 2016). This strategy not only provides a way to narrow-down known QTLs to candidate genes, but also delivers information regarding favorable natural SNP allelic variants of genes regulating complex quantitative traits in rice accessions that got domesticated and fixed by natural selection, and subsequently adapted in diverse agro-climatic conditions. Therefore, this combinatorial strategy can be instrumental for dissection of complex quantitative traits and marker-aided genetic enhancement to develop cultivars with high GWt and yield in rice (Ruggieri et al., 2014; Zhang et al., 2014; Kujur et al., 2015).

**FIGURE 1:**
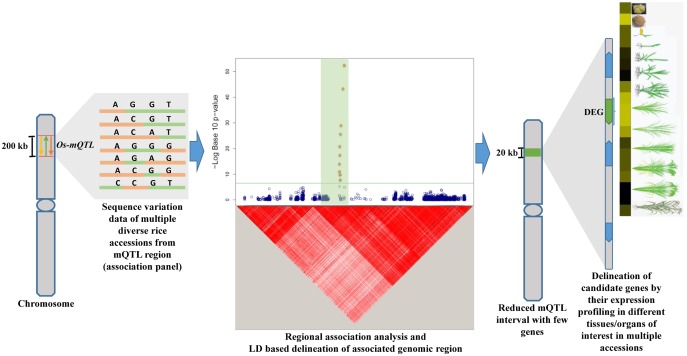
**Illustrative representation of an integrated genomic strategy combining mQTL analysis with regional association mapping and expression profiling for delineation of trait-associated potential genomic regions (genes) in rice.** Yellow, green, and orange color arrows depict different independently identified Quantitative trait loci (QTLs) for similar trait. The horizontal green line within Manhattan plot symbolizes significance threshold and green shaded region represents the associated region determined based on regional LD. The solid green bar on the chromosome depicts the reduced mQTL interval. DEG: differentially expressed gene are indicated by green color while other genes within the delineated metaQTL region are illustrated by blue color.

Keeping above prospects in view, the current study employed an integrated strategy combining traditional metaQTL analysis and regional association mapping (**Figure 1**) to validate and narrow-down long confidence intervals of six important metaQTL regions governing grain size/weight in Indian *indica* and aromatic rice accessions. This study will take us one step closer toward identifying functionally relevant potential genes/alleles underlying known grain size/weight QTLs mapped on Indian germplasm accessions-derived low-density genetic linkage maps. This will essentially assist us to select superior natural allelic variants from the potential QTLs/genes detected for grain size/weight and their effective deployment in yield improvement especially of Indian *indica* and aromatic rice accessions.

## Meta-Analysis Identifies Robust Genomic Regions Associated with Rice Grain Size/Weight

To identify genetic basis of grain size/weight variability in Indian *indica* and aromatic rice accessions, primarily the meta-analysis involving different QTL mapping studies was conducted. For this, extensive literature survey was conducted to prepare the comprehensive list of major grain size (GL, GWi, and GL/GWi) and GWt QTLs, which have been identified and mapped on different rice chromosomes using multiple preliminary and advanced generation mapping populations generated by inter-crossing among diverse Indian *indica* and aromatic rice accessions. The search resulted in identification of seven studies with 64 major/minor QTLs governing grain size and GWt traits mapped genetically on 12 rice chromosomes (Supplementary Table S1). The physical coordinates for the selected QTL intervals were defined by determining the physical positions (bp) of SSR (simple sequence repeat) markers flanking these QTLs as documented in RGAP (Rice Genome Annotation Project) pseudomolecule version 6.1^4^. Meta-analysis was further conducted using 64 identified grain size/weight QTLs in rice. For this, all these 64 QTLs were first projected on the reference rice genetic map and further meta-analysis was performed to detect robust metaQTLs on each chromosome using BioMercator V3 (Sosnowski et al., 2012) as per Deshmukh et al. (2012). Consequently, only those chromosomal metaQTL regions detected based on QTLs identified from at least two independent studies as well as with at least one of the QTL exhibiting LOD score ≥ 4 and phenotypic variance ≥ 10% were selected for further analysis. *GS3*, a well-known major grain size QTL interval served as a positive control and was also included as one of the metaQTL region (Fan et al., 2006).

**FIGURE 2:**
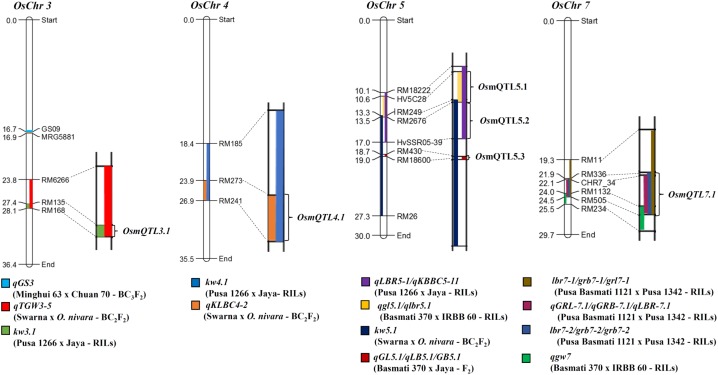
**Schematic diagram illustrating mQTL intervals selected for regional association analysis.** Different QTL intervals are depicted as shaded regions on chromosomes [color codes for each QTL is given at the bottom of each chromosome; the physical distance (Mb) and identity of the marker loci are indicated on the left and right side of each chromosome, respectively]. Chromosome regions harboring overlaps of two or more than two QTL intervals were selected for association analysis, marked as metaQTLs.

Meta-analysis of 64 grain size/weight QTLs (by integrating their genetic and corresponding physical positions) detected six different mQTL (metaQTLs) regions (with at least one of the overlapping QTL exhibited a LOD score ≥ 4 and phenotypic variance ≥ 10) that were mapped on four different rice chromosomes. The confidence marker intervals for these six mQTLs ranged from 0.33 to 3.48 Mb. The structural annotation of the identified mQTLs revealed the presence of various protein-coding genes ranging from 42 to 526. A mQTL mapped on chromosome 5 (*OsmQTL5.3*) was found to be the shortest, spanning 0.33 Mb with 42 genes whereas another mQTL located on same chromosome (*OsmQTL5.2*) was longest spanning 3.48 Mb containing 526 genes (**Table 1** and **Figure 2**). The efficacy of meta-analysis in refining the confidence intervals for previously known QTLs as well as for validating their effects across different genetic backgrounds and environments is well-demonstrated (Goffinet and Gerber, 2000; Wu and Hu, 2012). Summarily, meta-analysis of major grain size/GWt QTLs in our study effectively identified six promising mQTL regions that are validated in different genetic backgrounds and environments. Therefore, these mQTLs have potential to serve as most efficient candidate targets either for fine mapping or their direct utilization in MAS for rice genetic enhancement as suggested previously (Ballini et al., 2008; Courtois et al., 2009; Khowaja et al., 2009; Wang et al., 2010).

**Table 1:** Summary of metaQTL regions selected for association analysis.

metaQTL identities	Overlapping grain size/weight quantitative trait loci (QTLs)	Chromosomes	Start physical position (bp)	End physical position (bp)	Genomic intervals (bp)	Markers flanking metaQTLs	Number of genes within QTL intervals	References on QTL mapping studies
*OsmQTL3.1*	*kw3.1* and *qTGW3-5*	*OsChr3*	27411818	28091534	679716	RM135-RM168	108	Marathi et al., 2012; Swamy et al., 2012
*OsmQTL4.1*	*Kw4.1* and *qKBLC4-2*	*OsChr4*	23863414	26857374	2993960	RM273-RM241	500	Guleria et al., 2012;Swamy et al., 2012
*OsmQTL5.1*	*qLBR5-1, qKBBC5-1, qgl5.1, qlbr5.1*, and *kw5.1*	*OsChr5*	10600000	13480036	2880036	HV5C28-RM2676	416	Guleria et al., 2012;Swamy et al., 2012; Shanmugavadivel et al., 2013
*OsmQTL5.2*	*Kw5.1, qLBR5-1*, and *qKBBC5-1*	*OsChr5*	13480036	16962564	3482528	RM2676-HvSSR05-39	526	Guleria et al., 2012;Swamy et al., 2012
*OsmQTL5.3*	*Kw5.1, qGL5.1, qLB5.1*, and *GB5.1*	*OsChr5*	18691417	19018217	326800	RM430-RM18600	42	Swamy et al., 2012; Vemireddy et al., 2015
*OsmQTL7.1*	*Lbr7, grb7-1, grl7-1, qGRL-7.1, qgw7, lbr7-2, grb7-2*, and *grl7-2*	*OsChr7*	22127494	24527013	2399519	RM336-RM505	394	Amarawathi et al., 2008; Singh et al., 2012; Shanmugavadivel et al., 2013
*GS3*	*GS3* QTL (positive control)	*OsChr3*	16690372	16878706	188334	GS09-MRG5881	23	Fan et al., 2006


## An Optimized Combinatorial mQTL and Regional Association Mapping Strategy Efficiently Scales-Down A Known *GS3* Major QTL Region

To optimize regional association mapping, association analysis for each of six mQTL genomic regions was performed independently using the individual SNP- and haplotype-based methods implemented in GAPIT and hapQTL, respectively. For this, sequence variation (SNPs) data of 505 accessions belonging to three different rice populations (*indica*, *japonica*, and *aus*) for selected mQTL genomic regions were retrieved from RiceVarMap database^5^. SNPs as well as accessions with greater than 1% missing data or minor allele frequency (MAF) < 5% were discarded. Filtered high-quality SNPs and accessions were further considered for association analysis (Supplementary Table S1). The phenotyping data of three major grain size/weight-related traits (GL, GWi, and GWt) were measured in a panel of 505 diverse rice accessions belonging to *indica*, *japonica*, and *aus* populations. Wider phenotypic variation (GL: varying from 6.71 to 11.23 mm with a mean 8.56 mm, GWi: 1.98 to 3.44 mm with a mean 2.87 mm and GWt: 15.38 to 37.47 g with a mean 24.27 g) with normal frequency distribution for all three gain size/weight traits among selected rice accessions were observed. To begin with the regional association analysis was performed in a known mQTL region, which harbors *GS3*, a major gene regulating grain size especially in *indica* population, has been cloned and characterized earlier by fine mapping/map-based isolation, association mapping and functional genomic approaches (Fan et al., 2006, 2009; Mao et al., 2010). These previous studies demonstrated the significant role of a SNP allelic variant (C/A) causing non-synonymous amino acid substitution (serine to threonine) in a G-protein γ subunit gene delineated at a *GS3* QTL region in controlling long and short grain size differentiation in rice.

**FIGURE 3:**
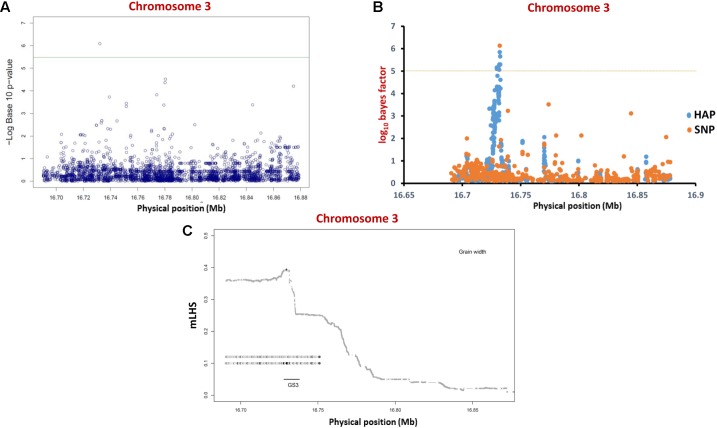
**Individual SNP- and haplotype-based regional association analysis of a known *GS3* QTL region. (A)** Individual SNP-based regional association analysis. The blue color circle depicts different SNPs whereas the green line represents significance threshold. **(B)** Haplotype-based association analysis. The orange circles represent single SNPs; the blue circles represent core SNPs within haplotypes (HAP) used for association. The horizontal dotted yellow line represents the significance threshold. **(C)** LD block (based on mLHS) around the most significant SNP (marked by “+”) is denoted with shaded lines. The top two ancestral haplotypes most significantly associated with grain length phenotype are designated as circles, with darker circles representing larger ancestral allele frequencies.

For regional association analysis, the SNP genotyping information and grain size/weight (GL, GWi, and GWt) phenotyping data of all 505 accessions as well as *indica* subset (365) of total 505 accessions were integrated using a CMLM interface of GAPIT independently. This analysis successfully revealed significant association (FDR corrected *P*-value of 0.002) of a known causal non-synonymous SNP (C/A at 16732087 bp on chromosome 3) reported previously in a G-protein γ subunit gene underlying a major *GS3* mQTL with grain size trait in *indica* population (but not in analysis with all 505 accessions) (**Figure 3A** and **Table 2**). The concerned SNP explained about 10% variation in GWi phenotype with A-allele positively affecting the grain size in rice. Like-wise, genetic association analysis was conducted for a *GS3* mQTL region for all three grain size/weight traits (GL, GWi, and GWt) using hapQTL (Xu and Guan, 2014), which employs haplotype-based strategy for establishing marker-trait association. This analysis identical to GAPIT detected significant association (Log_10_ BF of 6.51 with *P*-value 3.18) between G-protein γ subunit gene-derived known causal non-synonymous SNP (C/A at 16732087 bp on chromosome 3) underlying a *GS3* QTL with grain size in *indica* subpopulation (but not in analysis with all 505 accessions) (**Figure 3B** and **Table 3**). Subsequently, haplotype block was constructed around this significant trait-associated SNP that harbors five protein coding genes including the one coding for known G-protein γ subunit gene (**Figure 3C** and **Table 3**). These outcomes are in accordance with earlier rice grain size trait association mapping studies (Takano-Kai et al., 2009; Zhao et al., 2011; McCouch et al., 2016). Henceforth, individual and haplotype-based regional association mapping strategies adopted in our study have proficiency to detect more accurate and non-spurious marker-trait association for grain size/weight in rice. Consequently, this approach has potential in rapid delineation of functionally relevant genes and natural alleles underlying not only for known mQTL intervals (like *GS3* QTL interval) but also targeting diverse major/minor grain size/weight mQTLs for which major effect genes are unknown in rice.

**Table 2:** Summary of individual-SNP based regional association analysis in metaQTL intervals.

metaQTL identities	Chromosomes	Population group (number of accessions)	Traits associated	Most significant SNPs^∗^	Annotation	FDR corrected *p*-value	*R*^2^
*GS3*	*OsChr3*	*Indica* (365)	Grain width	sf0316732087	Intergenic	0.0025	0.101
*OsmQTL5.3*	*OsChr5*	*Indica, japonica*, and *aus* (505)	Grain length	sf0518872800	Intergenic	0.00093	0.059


*^∗^SNP IDs are same as used in RiceVarMap database*.

**Table 3:** Summary of haplotype-based regional association analysis in metaQTL intervals.

metaQTL identities	Chromosomes	metaQTL intervals (Mb)	Number of genes within metaQTL intervals	Population group (number of accessions)	Trait associated	Most significant SNPs^∗^	Structural annotation	BF2	P2	BF1	P1	Delineated metaQTL intervals (Mb)	Number of genes within delineated intervals
*GS3*	*OsChr3*	16.69–16.88 (0.19)	23	*Indica* (365)	Grain width	sf0316732087	Intergenic	5.85	3.18	6.14	3.89	16.66–16.75 (0.09)	5
*OsmQTL3.1*	*OsChr3*	27.41–28.09 (0.68)	108	*Indica, japonica*, and *aus* (505)	Grain width	sf0327811770	Intergenic	6.36	2.30	-0.19	0.66	27.68-27.93 (0.25)	34
*OsmQTL5.3*	*OsChr5*	18.69–19.01 (0.32)	42	*Indica, japonica* and *aus* (505)	Grain length	sf0518872800	Intergenic	5.01	2.08	4.482	4.76	18.84–18.97 (0.13)	12
*OsmQTL7.1*	*OsChr7*	22.13–22.53 (2.4)	394	*Indica, japonica*, and *aus* (505)	Grain length	sf0722700042	Intergenic	6.51	2.78	0.796	0.41	22.60–23.06 (0.46)	58
*OsmQTL3.1*	*OsChr3*	27.41–28.09 (0.68)	108	*Indica* (365)	Grain width	sf0327686607	Intergenic	5.81	2.03	0.138	0.50	27.67–27.70 (0.03)	4
*OsmQTL5.3*	*OsChr5*	18.69–19.01 (0.32)	42	*Indica* (365)	Grain weight	sf0518859830	Intergenic	3.0	0.98	5.17	2.40	18.72–18.95 (0.23)	27


*^∗^SNP IDs are same as used in RiceVarMap database*.

## Individual SNP-Based Regional Association Analysis Identifies Candidate Genes Underlying Grain Size/Weight mQTLs

For CMLM-based regional association analysis, high-quality SNP genotyping (of each selected mQTL genomic regions) and grain size/weight phenotyping (GL, GWi, and GWt) data for all 505 accessions (representing *indica*, *japonica*, and *aus* populations) were combined with their relative kinship matrix (K) and PCA [principal component (PC) analysis] information using P3D/compressed mixed linear model (CMLM) in GAPIT (Lipka et al., 2012; Kumar et al., 2015; Upadhyaya et al., 2015). The quantile-quantile plot generated in GAPIT output was employed to visualize relative distribution of observed and expected -log_10_ (*P*)-value for each SNP marker-trait association. The *P*-value threshold of significance was corrected for multiple comparisons based on false discovery rate (FDR cut-off ≤ 0.05) (Benjamini and Hochberg, 1995). Further, association analysis was performed using the genotyping and grain size/weight phenotyping data and diversity-related information (K and PCA with no PC covariates) of 365 accessions belonging to *indica* population following the aforesaid methods of GAPIT.

**FIGURE 4:**
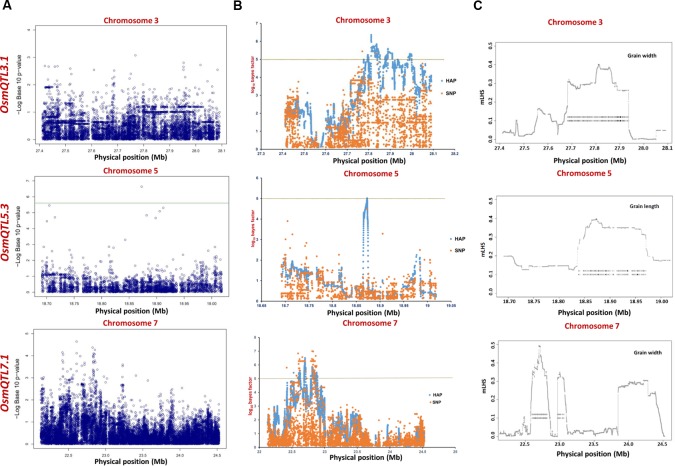
**Manhattan plots showing significant trait associations identified using all 505 accessions. (A)** Individual SNP-based regional association analysis, **(B)** haplotype-based regional association analysis and **(C)** trait-associated LD blocks calculated for each of the associated haplotype. The green and yellow lines represent the significance threshold.

**FIGURE 5:**
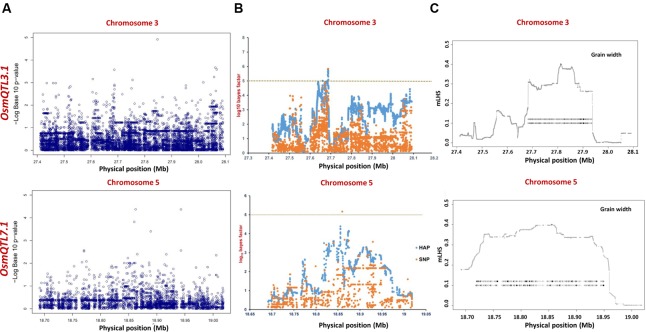
**Manhattan plots showing significant trait associations identified using 365 *indica* accessions. (A)** Individual SNP-based regional association analysis, **(B)** haplotype-based regional association analysis and **(C)** trait-associated LD blocks calculated for each of the associated haplotype. The green and yellow lines represent the significance threshold.

The integration of SNP genotyping data from six selected *OsmQTL* regions with grain size/weight-related phenotyping information (GL, GWi, and GWt) of all 505 accessions successfully detected significant association of a genomic locus underlying *OsmQTL5.3* with GL in rice (**Figure 4A** and **Table 2**). However, significant association could not be detected for any other *OsmQTL* regions with any of grain size/weight phenotypes when analysis was performed for *indica* subpopulation. A most significant SNP (G/T) (SNP ID: sf0518872800) within *OsmQTL5.3* was found to be located in the intergenic region between LOC_Os05g32430 (encoding NLI interacting factor-like phosphatase protein) and LOC_Os05g32440 (unknown expressed protein). Considering the known estimated average LD decay rate in rice (Huang et al., 2010), 20 protein-coding genes which lie within 100 kb up- and down-stream regions of a most significant SNP (G/T) (at 18872800 bp on a chromosome 5) were considered as putative candidates in regulating rice GL. None of the genomic loci underlying the rest five *OsmQTL* regions exhibited significant association with any of the three grain size/weight traits, when analyzed in all 505 accessions or 365 *indica* subpopulation (**Figures 4A, 5A** and **Table 2**).

## Haplotype-Based Regional Association Analysis Detects Novel Associations at Grain Size/Weight mQTLs

Haplotype-based regional association analysis was performed by integrating high-quality SNP (for each of the selected mQTL genomic regions) genotyping data with grain size/weight phenotyping information (GL, GWi, and GWt) of all 505 rice accessions belonging to three different rice populations (*indica, japonica*, and *aus*) using hapQTL. To determine population structure, PCA was performed with filtered high-quality SNPs using TASSEL 5.0 and top three PCs were used as covariates in regression model during analysis. Threshold for significance BF (Bayesian factor) was selected as maximum log_10_ BF (bayes factor) under null for all three grain size/weight phenotypes combined plus one (Xu and Guan, 2014). In entire analysis, number of upper level clusters (S), which represent source population were set to 3 and lower level clusters (K) signifying the ancestral haplotypes, were set to 10. Multiple (10) EM (expectation-maximization) runs were used to avoid uncertainty in inferring LD. LD blocks around the most significant associations were quantified by computing mLHS (local haplotype sharing between SNP markers) between the most significant SNP marker loci and the all remaining SNPs. Largest genomic regions, which have an mLHS value 2.5 times greater than background mLHS (i.e., 0.25 for *K* = 10) value, were defined as trait-associated LD blocks. Like-wise, haplotype-based association analysis (with no PC covariates) was performed independently by integrating genotyping data with grain size/weight phenotyping information of 365 *indica* accessions as per aforementioned methods.

The haplotype-based association analysis of six selected *OsmQTL* regions using hapQTL in all 505 accessions detected significant association of *OsmQTL5.3* (SNP T/G at 18872800 bp on chromosome 5 with log_10_ BF of 5.02 for the haplotype) with GL (**Figure 4B** and **Table 3**). The outcome is agreed-well with our aforementioned CMLM-based association analysis with all 505 accessions, thereby further augmenting the authenticity of detected marker-trait association in rice. In addition, hapQTL analysis detected association of *OsmQTL3.1* (SNP G/A at 327811770 bp on chromosome 3 with log_10_ BF of 6.36 for haplotype) and *OsmQTL7.1* (SNP A/G at 22700042 bp on chromosome 7 with log_10_ BF of 6.51 for haplotype) with GWi and GL, respectively. Subsequent analysis with *indica* subpopulation could only detect significant association of *OsmQTL3.1* (SNP T/C at 27686607 bp on chromosome 3 with log_10_ BF of 5.81 for the haplotype) with GWi and *OsmQTL5.3* (SNP G/A at 18859830 bp on chromosome 5 with log_10_ BF of 5.17 for the haplotype) with GWt (**Figure 5B** and **Table 3**). Interestingly, two additional novel marker-trait associations (detected both in *indica* subpopulations and in all 505 accessions) aside those identified using GAPIT were detected using hapQTL (**Figures 4A,B**). This highlights the power of haplotype-led association strategies in detecting novel associations, which are difficult to detect with individual SNP-based association methods due to its inability to deal with allelic as well as locus heterozygosity (Xu and Guan, 2014). The mLHS was further quantified around core SNPs (of most significant haplotypes) to determine the LD blocks (trait-associated regions) harboring the candidate genes associated with the analyzed grain size/weight trait. The LD block quantified using all 505 accessions around the most significant SNP (MSP) within *OsmQTL3.1* (G/A at 327811770 bp on chromosome 3) spanned from 27.68 to 27.94 Mb containing 34 genes whereas those calculated around the MSP within *OsmQTL5.3* (T/G at 18872800 bp on chromosome 5) and *OsmQTL7.1* extended from 18.84 to 18.97 Mb and 22.60 to 23.06 Mb harboring 12 and 58 rice genes, respectively (**Figure 4C** and **Table 3**). Like-wise, LD bocks were also estimated around MSPs detected with *indica* subpopulation using 365 *indica* accessions. The LD block around MSP detected within *OsmQTL3.1* (T/C at 27686607 bp on chromosome 3) extended from 27.67 to 27.71 Mb with only four genes, whereas those around MSP detected within *OsmQTL5*.3 (T/G at 18872800 bp on chromosome 5) spanned from 18.72 to 18.95 Mb with 37 genes (**Figure 5C** and **Table 3**).

Summarily, haplotype-based association analysis and subsequent LD block quantification using genotyping data of all 505 accessions significantly narrowed-down original confidence marker intervals for *OsmQTL3.1*, *OsmQTL5.3*, and *OsmQTL7.1* from 0.68 (108 genes), 0.33 (42 genes), and 2.40 (394 genes) Mb to 0.25 (34 genes), 0.13 (12 genes), and 0.46 (58 genes) Mb, respectively (**Figure 4** and **Table 3**). In case of similar analysis with *indica* subpopulation, the intervals for *OsmQTL3*.1 and *OsmQTL5.3* were scaled-down from 2.4 (108 genes) and 0.33 (42 genes) Mb to 0.03 (4 genes) and 0.23 (27 genes) Mb, respectively (**Figure 4** and **Table 3**). In case of *OsmQTL5.3*, mLHS calculation with all 505 accessions produced smaller LD block as compared to those calculated with 365 *indica* accessions, showing evidence of inverse correlation between number of accessions used for mLHS calculation and size of LD block as suggested previously (Xu and Guan, 2014). However, in case of *OsmQTL3.1*, the LD block was found much smaller in *indica* analysis, which can be explained by lower LD in *indica* subset (365 accessions) (McCouch et al., 2016). This provides further opportunity to narrow-down these mQTL regions by increasing the number of germplasm accessions for haplotype-based association analysis and subsequent quantification of LD blocks as well as through subpopulation-specific association analysis. Moreover, the mLHS-based quantification of LD blocks has a greater potential to scale-down the longer mQTL intervals into shorter genomic intervals as compared to *R* square-based quantification used traditionally for association analysis. Therefore, haplotype-based regional association analysis has profound efficacy for rapid delineation of candidate genes and natural alleles underlying major QTLs in rice (Xu and Guan, 2014).

## *In Silico* Global Expression Profiling Delineates Candidate Genes at mQTL Intervals Narrowed-Down by Regional Association Analysis

Integration of expression profiling-based functional evidences with association analysis is very popular approach for further delineating candidate genes within associated LD blocks and has been successfully employed in variety of crops (Das et al., 2016). In order to further prioritize candidate genes delineated by regional association mapping, global *in silico* expression profiling of these genes were performed utilizing the whole genome microarray profiling data for diverse vegetative and reproductive stages of Nipponbare cultivar available at RiceXPro database^6^. This includes microarray expression profiles of different tissues such as total inflorescence, floral organs (anther, pistil, lemma, and palea) at early reproductive development stages, embryo and endosperm during early grain development stages as well as seed maturation stages.

Expression profiles of all candidate genes within delineated mQTL regions (validated and narrowed-down by regional association mapping) were compared with aforesaid various vegetative and reproductive tissues using global *in silico* expression analysis. Most of the genes displayed differential expression when compared between various tissues at different stages of reproductive development. Some of the genes within the delineated genomic intervals were found to express at very high levels in spikelet hull (lemma and palea) during early reproductive development stages and therefore, can be involved in spikelet hull development and essentially in seed development. For instance, one gene (LOC_Os05g32460) delineated within reduced *OsmQTL5.3* region (0.1 Mb), which codes for VQ domain-containing protein and another gene (LOC_Os03g48660) located within reduced *OsmQTL3.1* interval (0.13 Mb) encoding DUF966 protein were found to be highly expressed in both lemma and palea during early stages of seed development and in endosperm during seed maturation (Supplementary Figure S1). Interestingly, the genes encoding VQ-domain containing proteins have been previously reported to be involved in seed development and maturation in plants (Wang et al., 2010; Jing and Lin, 2015). The orthologs of DUF966 protein coding gene (LOC_Os03g48660) are known to be regulated by auxin, which have well established role in seed development along with other hormones (Locascio et al., 2014). Therefore, both of these genes can be considered as most promising candidates for further functional validation.

Collectively, the outcomes of the present study indicate the potential utility of high-resolution association mapping in reduction of grain size/weight mQTL intervals derived from diverse QTL mapping studies in rice. The reduced grain size/weight major mQTLs can be directly utilized in MAS for rice genetic improvement given their consistent effects in different genetic backgrounds and environments. Moreover, two potential candidate genes exhibiting similarity with earlier characterized *Arabidopsis* known grain size/weight orthologs (Locascio et al., 2014), delineated by us employing an integrated strategy (mQTL region-specific association analysis and expression profiling) can further be used for functional validation studies and translational genomic analyses for rice genetic enhancement. With the availability of huge sequencing- and array-based SNP genotyping information for numerous phenotypically well-characterized germplasm accessions of major food crops such as maize, wheat, soybean etc., the developed strategy can be readily extended to these crop plants for identification of valid candidate genes and natural alleles associated with important agronomic traits. This essential information will be of immense use in quick molecular genetic dissection of complex quantitative traits to expedite genomics-assisted crop improvement.

## Author Contributions

AD written the manuscript and performed all analysis. RS helped in data analysis. AS, SP, and AT conceptualized and guided the study.

## Conflict of Interest Statement

The authors declare that the research was conducted in the absence of any commercial or financial relationships that could be construed as a potential conflict of interest.

## Footnotes

1 http://www.gramene.org

2 http://qtaro.abr.affrc.go.jp

3 http://irri.org/our-work/research/genetic-diversity/international-rice-genebank

4 http://rice.plantbiology.msu.edu/pub/data/Eukaryotic_Projects/o_sativa/annotation_dbs/pseudomolecules/version_6.1/

5 http://ricevarmap.ncpgr.cn/

6 http://ricexpro.dna.affrc.go.jp/
