# Supplementary material for: Regional Association Analysis of MetaQTLs Delineates Candidate Grain Size Genes in Rice
Source: Front Plant Sci. 2017 May 29;8:807. doi: 10.3389/fpls.2017.00807 (PMC5447001; doi:10.3389/fpls.2017.00807)
Supplement: Supplementary file 1 [file Table_1.pdf]

Table S1. Summary of grain size/weight QTLs used for meta analysis in rice

QTL indentities	Traits	Chromosomes	Left flanking primers	Right flanking primers	Start physical positions (bp)	End physical positions(bp)	Physical intervals (bp)	*LOD	*R^2	Mapping populations	Population types	References
grb7-2	Grain breadth	7	RM505	RM336	24527013	21871201	2655812	9.2	0.189	Pusa Basmati 1121 × Pusa 1342	RIL	Amarawathi et al. (2008)
grl7-2	Grain length	7	RM505	RM336	24527013	21871201	2655812	3.02	0.057	Pusa Basmati 1121 × Pusa 1342	RIL	Amarawathi et al. (2008)
lbr7-2	Length/breadth ratio	7	RM505	RM336	24527013	21871201	2655812	10.8	0.219	Pusa Basmati 1121 × Pusa 1342	RIL	Amarawathi et al. (2008)
grb7-1	Grain breadth	7	RM11	RM505	19256791	24527013	5270222	6.15	0.101	Pusa Basmati 1121 × Pusa 1342	RIL	Amarawathi et al. (2008)
grl7-1	Grain length	7	RM11	RM505	19256791	24527013	5270222	4.05	0.074	Pusa Basmati 1121 × Pusa 1342	RIL	Amarawathi et al. (2008)
lbr7-1	Length/breadth ratio	7	RM11	RM505	19256791	24527013	5270222	6.2	0.1	Pusa Basmati 1121 × Pusa 1342	RIL	Amarawathi et al. (2008)
grl 1-1	Grain length	1	RM431	RM104	38893896	40166840	1272944	5.75	0.101	Pusa Basmati 1121 × Pusa 1342	RIL	Amarawathi et al. (2008)
qKLBC4-1	Kernel length	4	RM307	RM401	13141966	13154288	12322	3.03	0.029	Pusa 1266 × Jaya	RIL	Guleria et al. (2012)
qKLBC4-2	Kernel length	4	RM273	RM241	23863414	26857374	2993960	3.81	0.05	Pusa 1266 × Jaya	RIL	Guleria et al. (2012)
qKBBC3-1	Kernel breadth	3	RM569	RM5474	1908094	3803115	1895021	3.78	0.072	Pusa 1266 × Jaya	RIL	Guleria et al. (2012)
qKBBC5-1	Kernel breadth	5	RM18222	HvSSR05-39	10123637	16962564	6838927	6.76/10.32	0.135/21.7	Pusa 1266 × Jaya	RIL	Guleria et al. (2012)
qKBBC8-1	Kernel breadth	8	RM25	GNMS2765	34032918	66408	33966510	3.62	0.127	Pusa 1266 × Jaya	RIL	Guleria et al. (2012)
qLBR5-1	Length/breadth ratio	5	RM18222	HvSSR05-39	10123637	16962564	6838927	9.86/5.80	0.15/0.129	Pusa 1266 × Jaya	RIL	Guleria et al. (2012)
qLBR12-1	Length/breadth ratio	12	GNMS3766	GNMS3781	75398	18185352	18109954	2.94	0.31	Pusa 1266 × Jaya	RIL	Guleria et al. (2012)
qTGW3-1	1000 grain weight	3	GNMS1289	RM5474	274626	3803115	3528489	8.08	0.13	Pusa 1266 × Jaya	RIL	Marathi et al. (2011)
qTGW3-2	1000 grain weight	3	RM3766	RM157A	6933576	9491727	2558151	3.33	0.05	Pusa 1266 × Jaya	RIL	Marathi et al. (2011)
qTGW3-3	1000 grain weight	3	GNMS1140	RM15283	27231835	18714975	8516860	5.23	0.06	Pusa 1266 × Jaya	RIL	Marathi et al. (2011)
qTGW3-4	1000 grain weight	3	RM3698	RM16	21783250	23126109	1342859	5.42	0.07	Pusa 1266 × Jaya	RIL	Marathi et al. (2011)
qTGW3-5	1000 grain weight	3	RM6266	RM168	23821947	28091534	4269587	6.08	0.1	Pusa 1266 × Jaya	RIL	Marathi et al. (2011)
qTGW4-1	1000 grain weight	4	RM3276	RM1112	30496500	34236058	3739558	4.76/3.81	0.07/0.05	Pusa 1266 × Jaya	RIL	Marathi et al. (2011)
qTGW5-1	1000 grain weight	5	GNMS1776	RM413	2146553	2212742	66189	4.93/5.11	0.18/0.2	Pusa 1266 × Jaya	RIL	Marathi et al. (2011)
qTGW6-1	1000 grain weight	6	RM6273	RM204	130368	3168545	3038177	12.9/6.39	0.25/014	Pusa 1266 × Jaya	RIL	Marathi et al. (2011)
qTGW9-1	1000 grain weight	9	RM278	RM160	19320040	19788238	468198	2.92	0.04	Pusa 1266 × Jaya	RIL	Marathi et al. (2011)
qTGW12-1	1000 grain weight	12	RM1261	GNMS3781	17531111	18185352	654241	2.77	0.07	Pusa 1266 × Jaya	RIL	Marathi et al. (2011)
qTGW12-2	1000 grain weight	12	RM1103	RM17	23539441	26954700	3415259	4.23/7.22/3.03	0.1/0.18/0.07	Pusa 1266 × Jaya	RIL	Marathi et al. (2011)
qgb7.2	Grain breadth	7	RM3831	RM1353	1164715	3311766	2147051	2.19	0.039	Basmati 370 × IRBB 60	RIL	Shanmugavadivel et al. (2013)
qgl11.1	Grain length	11	HV11C13	RM26279	5800000	6348705	548705	3.18	0.057	Basmati 370 × IRBB 60	RIL	Shanmugavadivel et al. (2013)
qlbr11.1	Length/breadth ratio	11	HV11C13	RM26279	5800000	6348705	548705	2.93	0.048	Basmati 370 × IRBB 60	RIL	Shanmugavadivel et al. (2013)
qgb1.2	Grain breadth	1	RM8123	RM3412	9239723	11583024	2343301	5.05/2.31	0.112/0.056	Basmati 370 × IRBB 60	RIL	Shanmugavadivel et al. (2013)
qlbr1.3	Length/breadth ratio	1	RM8123	RM3412	9239723	11583024	2343301	3.95/2.83	0.086/0.065	Basmati 370 × IRBB 60	RIL	Shanmugavadivel et al. (2013)
qgl5.1	Grain length	5	HV5C28	RM2676	10600000	13480036	2880036	2.92/3.11	0.048/0.071	Basmati 370 × IRBB 60	RIL	Shanmugavadivel et al. (2013)
qlbr5.1	Length/breadth ratio	5	HV5C28	RM2676	10600000	13480036	2880036	2.88/3.69	0.058/0.078	Basmati 370 × IRBB 60	RIL	Shanmugavadivel et al. (2013)
qgb7.1	Grain breadth	7	RM1353	RM1279	3311766	21613971	18302205	2.39	0.116	Basmati 370 × IRBB 60	RIL	Shanmugavadivel et al. (2013)
qgb1.3	Grain breadth	1	RM129	RM8144	19008692	23208983	4200291	2.73	0.059	Basmati 370 × IRBB 60	RIL	Shanmugavadivel et al. (2013)
qgl1.4	Grain length	1	RM129	RM8144	19008692	23208983	4200291	3.79	0.128	Basmati 370 × IRBB 60	RIL	Shanmugavadivel et al. (2013)
qlbr1.4	Length/breadth ratio	1	RM129	RM8144	19008692	23208983	4200291	3.92/2.86	0.103/0.106	Basmati 370 × IRBB 60	RIL	Shanmugavadivel et al. (2013)
qgw7	Grain weight	7	RM1132	RM234	23984500	25472630	1488130	2.6	0.052	Basmati 370 × IRBB 60	RIL	Shanmugavadivel et al. (2013)
qgb8	Grain breadth	8	RM80	RM4997	24478642	28235762	3757120	6.46/5.27	0.243/0.205	Basmati 370 × IRBB 60	RIL	Shanmugavadivel et al. (2013)
qgw8	Grain weight	8	RM80	RM4997	24478642	28235762	3757120	4.1/9.53	0.092/0.326	Basmati 370 × IRBB 60	RIL	Shanmugavadivel et al. (2013)
qlbr8	Length/breadth ratio	8	RM80	RM4997	24478642	28235762	3757120	2.85/2.43	0.14/0.069	Basmati 370 × IRBB 60	RIL	Shanmugavadivel et al. (2013)
qgl2	Grain length	2	RM1303	RM318	20966763	29631532	8664769	10.02	0.133	Basmati 370 × IRBB 60	RIL	Shanmugavadivel et al. (2013)
qgb1.4	Grain breadth	1	RM8144	RM302	23208983	32968583	9759600	2.47	0.054	Basmati 370 × IRBB 60	RIL	Shanmugavadivel et al. (2013)
qgl1.1	Grain length	1	RM8144	RM302	23208983	32968583	9759600	12.49/10.31	0.179/0.166	Basmati 370 × IRBB 60	RIL	Shanmugavadivel et al. (2013)
qlbr	Length/breadth ratio	1	RM8144	RM302	23208983	32968583	9759600	5.94/6.89	0.159/0.24	Basmati 370 × IRBB 60	RIL	Shanmugavadivel et al. (2013)
qGRB-7.1	Grain breadth	7	CHR7_34	RM505	22127494	24527013	2399519	2.95	0.086	Pusa Basmati 1121 × Pusa 1342	RIL	Singh et al. (2012)
qGRL-7.1	Grain length	7	CHR7_34	RM505	22127494	24527013	2399519	3.18	0.152	Pusa Basmati 1121 × Pusa 1342	RIL	Singh et al. (2012)
qLBR-7.1	Length/breadth ratio	7	CHR7_34	RM505	22127494	24527013	2399519	2.95	0.086	Pusa Basmati 1121 × Pusa 1342	RIL	Singh et al. (2012)
qGRL-1.1	Grain length	1	CHR1_1	RM431	38785913	38893896	107983	2.87	0.108	Pusa Basmati 1121 × Pusa 1342	RIL	Singh et al. (2012)
kw8.1	Kernel width	8	RM152	RM38	683117	2114843	1431726	4.2	13	Swarna × *Oryza nivara* [IRGC81832]	BC_2F_2	Swamy et al. (2012)
lwr12.1	Length/breadth ratio	12	RM415	RM19	426173	2432228	2006055	2.56	0.9	Swarna × *Oryza nivara* [IRGC81832]	BC_2F_2	Swamy et al. (2012)
kw1.1	Kernel width	1	RM499	RM428	388856	2606778	2217922	3.3	0.3	Swarna × *Oryza nivara* [IRGC81832]	BC_2F_2	Swamy et al. (2012)

QTL indentities	Traits	Chromosomes	Left flanking primers	Right flanking primers	Start physical positions (bp)	End physical positions(bp)	Physical intervals (bp)	*LOD	*R^2	Mapping populations	Population types	References
kl1.1	Kernel length	1	RM499	RM84	388856	5647816	5258960	3.3	0.6	Swarna × *Oryza nivara* [IRGC81832]	BC_2F_2	Swamy et al. (2012)
lwr2.1	Length/breadth ratio	2	RM174	RM324	7006085	11389941	4383856	2.7	0.7	Swarna × *Oryza nivara* [IRGC81832]	BC_2F_2	Swamy et al. (2012)
kw6.1	Kernel width	6	RM204	RM3	3168374	19499522	16331148	3.2	0.12	Swarna × *Oryza nivara* [IRGC81832]	BC_2F_2	Swamy et al. (2012)
kw4.1	Kernel width	4	RM185	RM241	18422098	26857374	8435276	2.9	0.12	Swarna × *Oryza nivara* [IRGC81832]	BC_2F_2	Swamy et al. (2012)
kw5.1	Kernel width	5	RM249	RM26	13270464	27341978	14071514	4.2	0.9	Swarna × *Oryza nivara* [IRGC81832]	BC_2F_2	Swamy et al. (2012)
kw3.1	Kernel width	3	RM135	RM168	27411818	28091534	679716	2.8	0.17	Swarna × *Oryza nivara* [IRGC81832]	BC_2F_2	Swamy et al. (2012)
kw6.1	Kernel width	6	RM190	RM314	1764563	29631532	27866969	2.85	0.9	Swarna × *Oryza nivara* [IRGC81832]	BC_2F_2	Swamy et al. (2012)
kw1.4	Kernel width	1	RM226	RM431	34032887	38893896	4861009	4.4	0.12	Swarna × *Oryza nivara* [IRGC81832]	BC_2F_2	Swamy et al. (2012)
qGB8.1	Grain breadth	8	RM502	RM310	26492216	5115740	21376476	3.454	0	Basmati 370 × Jaya	F_2	Vemireddy et al. (2015)
qGB5.1	Grain breadth	5	RM430	RM18600	18691417	19018217	326800	3.333	0.171	Basmati 370 × Jaya	F_2	Vemireddy et al. (2015)
qGL5.1	Grain length	5	RM430	RM18600	18691417	19018217	326800	6.603	0.217	Basmati 370 × Jaya	F_2	Vemireddy et al. (2015)
qLB5.1	Length/breadth ratio	5	RM430	RM18600	18691417	19018217	326800	4.65	0.465	Basmati 370 × Jaya	F_2	Vemireddy et al. (2015)

*LOD and R^2 of QTLs detected across multiple years are separated by slash
