# Supplementary figures and images for: Regional Association Analysis of MetaQTLs Delineates Candidate Grain Size Genes in Rice
Source: Front Plant Sci. 2017 May 29;8:807. doi: 10.3389/fpls.2017.00807 (PMC5447001; doi:10.3389/fpls.2017.00807)

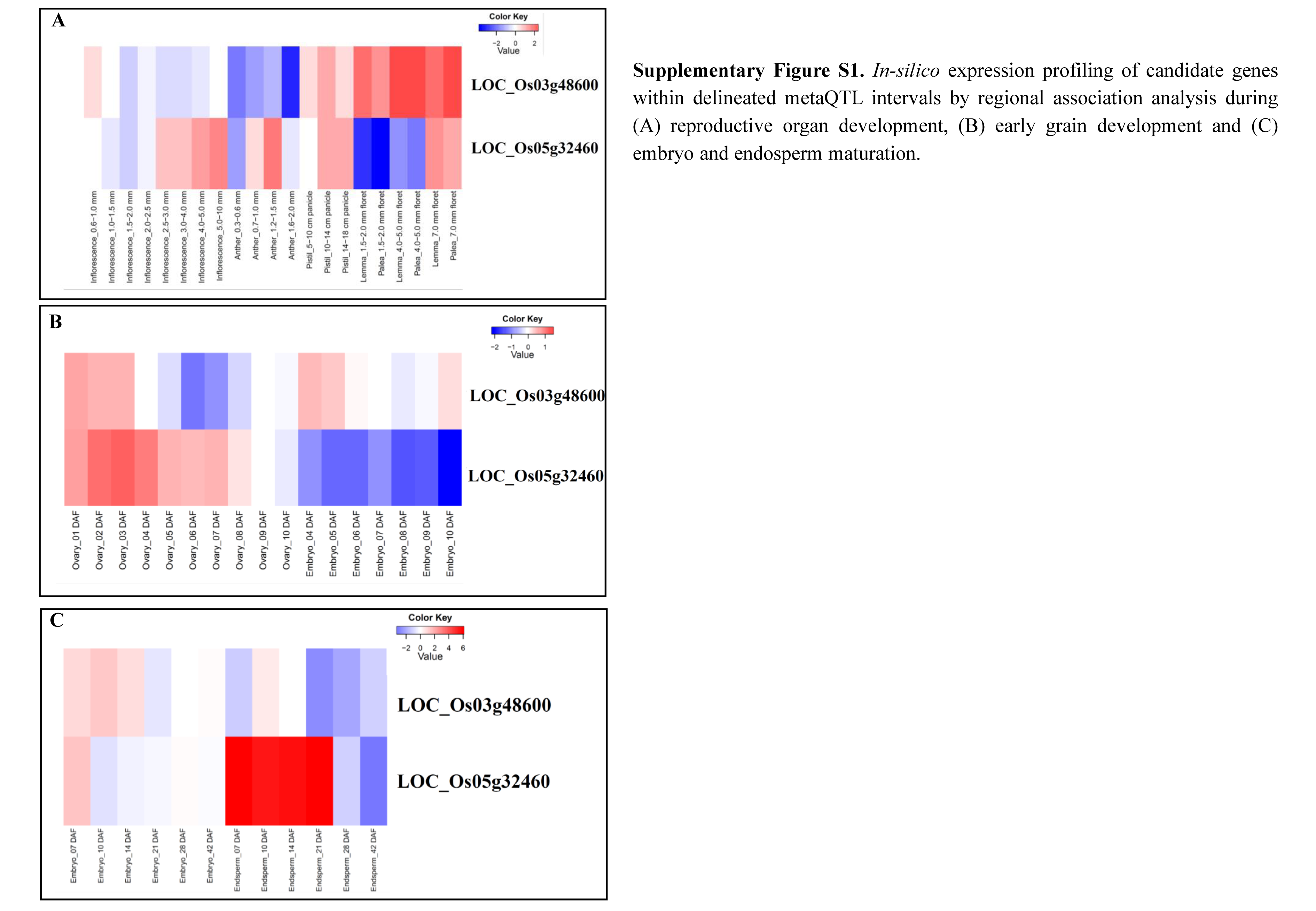

Supplement: Supplementary file 2 [file Image_1.TIF]
